# Antigenic variants of influenza B viruses isolated in Japan during the 2017‐2018 and 2018‐2019 influenza seasons

**DOI:** 10.1111/irv.12713

**Published:** 2020-01-19

**Authors:** Sari Kato‐Miyashita, Yuko Sakai‐Tagawa, Makoto Yamashita, Kiyoko Iwatsuki‐Horimoto, Mutsumi Ito, Akifumi Tokita, Haruhisa Hagiwara, Naomi Izumida, Tamon Nishino, Noriyuki Wada, Michiko Koga, Eisuke Adachi, Daisuke Jubishi, Hiroshi Yotsuyanagi, Yoshihiro Kawaoka, Masaki Imai

**Affiliations:** ^1^ Division of Virology Department of Microbiology and Immunology Institute of Medical Science University of Tokyo Tokyo Japan; ^2^ Clinic Bambini Tokyo Japan; ^3^ Members of the Tokyo Pediatric Association Public Health Committee Tokyo Japan; ^4^ Hagiwara Clinic Tokyo Japan; ^5^ Akebonocho Clinic Tokyo Japan; ^6^ Alpaca Kids Ent Clinic Tokyo Japan; ^7^ Wada Pediatric Clinic Tokyo Japan; ^8^ Division of Infectious Diseases Advanced Clinical Research Center Institute of Medical Science University of Tokyo Tokyo Japan; ^9^ Department of Infectious Diseases and Applied Immunology IMSUT Hospital of the Institute of Medical Science University of Tokyo Tokyo Japan; ^10^ Nezu Clinic Tokyo Japan; ^11^ Department of Pathobiological Sciences School of Veterinary Medicine Influenza Research Institute University of Wisconsin Madison WI USA; ^12^ Department of Special Pathogens International Research Center for Infectious Diseases Institute of Medical Science University of Tokyo Tokyo Japan

**Keywords:** antigenicity, hemagglutinin, influenza B virus, Japan, neuraminidase inhibitors

## Abstract

**Background:**

Here, we genetically and antigenically analyzed influenza B viruses (IBVs) isolated in Japan during the 2017‐2018 and 2018‐2019 influenza seasons.

**Methods:**

A total of 68 IBVs (61 B/Yamagata/16/88‐like [B/Yamagata]‐lineage and 7 B/Victoria/2/87‐like [B/Victoria]‐lineage) were antigenically and genetically characterized by using hemagglutination inhibition (HI) assays and phylogenetic analysis, respectively. The susceptibility of IBVs to neuraminidase (NA) inhibitors was assessed by using a fluorescence‐based NA inhibition assay.

**Results:**

All 61 B/Yamagata‐lineage isolates were genetically closely related to B/Phuket/3073/2013, the vaccine strain for these two seasons. Eleven B/Yamagata‐lineage isolates tested were antigenically similar to B/Phuket/3073/2013 by the HI test. Seven B/Victoria‐lineage isolates were genetically closely related to B/Texas/02/2013, the WHO‐recommended vaccine strain for the 2017‐2018 season; however, they were antigenically distinct from B/Texas/02/2013 with an eightfold or 16‐fold difference in HI titer. Of these 7 isolates, 4 possessed a two‐amino‐acid deletion at positions 162 and 163 in hemagglutinin (HA) and the other 3 had a three‐amino‐acid deletion at positions 162‐164 in HA. Importantly, the variants with the three‐amino‐acid deletion appeared to be antigenically different from the B/Colorado/06/2017 virus with the two‐amino‐acid deletion, the vaccine strain for the 2018‐2019 season with a fourfold or eightfold difference in HI titer. One B/Yamagata‐lineage isolate carrying a G407S mutation in its NA showed a marked reduction in susceptibility to zanamivir, peramivir, and laninamivir.

**Conclusions:**

These results highlight the need for continued monitoring for the prevalence of the antigenic variant with the three‐amino‐acid deletion and the variant with reduced NA inhibitor susceptibility.

## 1 INTRODUCTION

Influenza is an acute respiratory infectious disease caused by influenza A and B viruses. Currently, two subtypes of influenza A viruses (A/H1N1 and A/H3N2) and two lineages of influenza B viruses (B/Victoria/2/87‐like [B/Victoria]‐ and B/Yamagata/16/88‐like [B/Yamagata]‐lineage) are cocirculating in the human population and cause epidemics of seasonal influenza. Globally, each year, an estimated 5%‐10% of adults and 20%‐30% of children are infected with seasonal influenza viruses, causing 3‐5 million cases of severe illness,^1^ and an estimated 290 000‐650 000 influenza‐related deaths.^2^ Therefore, seasonal influenza is associated with significant morbidity and mortality worldwide.

The influenza A and B viruses possess two surface glycoproteins, hemagglutinin (HA) and neuraminidase (NA). HA is the major antigen, which elicits the production of neutralizing antibodies by the host after infection or vaccination. The accumulation of point mutations in the antigenic sites of HA enables viruses to evade host immune responses induced by prior infections or vaccinations, resulting in the emergence of new antigenic variants with epidemic potential.^3^, ^4^ Anti‐influenza drugs that inhibit the enzymatic activity of NA are available for the treatment and prophylaxis of influenza; however, mutations in the NA active site reduce its susceptibility to NA inhibitor drugs, leading to the emergence of drug‐resistant variants.^5^, ^6^

During the 2017‐2018 influenza season, influenza A/H1N1 2009 pandemic (A/H1N1pdm), A/H3N2, and B viruses co‐circulated in Japan.^7^ Notably, in Japan, the influenza B epidemic of this season was larger than that of the previous nine seasons. Here, we examined the genetic and antigenic properties of the influenza B viruses isolated in Japan during the winter season. In addition, we characterized the influenza B viruses isolated in Japan during the 2018‐2019 season, even though influenza B viruses circulated at a lower level in this season compared with the previous season.

## 2 MATERIALS AND METHODS

### 2.1 Clinical specimens

After informed consent was obtained, respiratory specimens were collected from patients with influenza‐like symptoms who visited clinics in Tokyo, Japan during the 2017‐2018 and 2018‐2019 seasons. For pediatric patients, informed consent was obtained from the parents. The specimens were submitted to the Division of Virology, Department of Microbiology and Immunology, Institute of Medical Science, the University of Tokyo for virus isolation. The research protocol was approved by the Research Ethics Review Committee of the Institute of Medical Science of the University of Tokyo (approval no. 26‐42‐0822). Samples that were influenza B‐positive by real‐time RT‐PCR (see below) were used in this study.

### 2.2 Cells

Madin‐Darby canine kidney (MDCK) and AX4^8^ cells were maintained in Eagle's minimal essential media (MEM) containing 5% newborn calf serum (NCS). hCK cells were maintained in the presence of 2 µg/mL puromycin and 10 µg/mL blasticidin in MEM containing 5% NCS.^9^ Human embryonic kidney 293T cells were maintained in Dulbecco's modified Eagle's medium containing 10% fetal calf serum. The cells were incubated at 37°C with 5% CO_2_ and regularly tested for mycoplasma contamination by using PCR and were confirmed to be mycoplasma‐free.

### 2.3 Virus isolation and propagation

MDCK, AX4, or hCK cells grown in 12‐well plates were inoculated with 0.1 mL per well of the clinical samples and incubated at 33°C for at least 30 minutes. One milliliter of MEM containing 0.3% bovine serum albumin (BSA) and 1 µg/mL TPCK‐treated trypsin was then added to cells. The cultures were then incubated for up to 7 days, until cytopathic effects were evident. Cell culture supernatants were harvested, viral RNA was extracted and subjected to RT‐PCR, and the viral genes were sequenced (see below). Influenza B viruses were propagated in MDCK, AX4, or hCK cells in MEM containing 1 µg of L‐1‐Tosylamide*‐*2*‐*phenylethyl chloromethyl ketone (TPCK)‐trypsin/mL at 33°C.

### 2.4 Reverse genetics

NA inhibitor‐sensitive and NA inhibitor‐resistant control viruses were generated by using a plasmid‐based reverse genetics system as previously described.^10^ In brief, plasmids encoding the complementary DNAs for the eight viral RNA segments under the control of the human RNA polymerase I promoter and the mouse RNA polymerase I terminator (referred to as PolI plasmids), and plasmids for the expression of the viral PB2, PB1, PA, and nucleoprotein proteins derived from influenza B virus strain B/Yamagata/1/73, under the control of the chicken β‐actin promoter,^11^ were transfected into 293T cells with the help of a transfection reagent, Trans‐IT 293 (Mirus). At 48‐hours post‐transfection, culture supernatants were collected and inoculated to MDCK cells for virus propagation. All virus stocks were sequenced to confirm the absence of unwanted mutations.

### 2.5 Real‐time RT‐PCR

Real‐time PCR was performed to determine the lineage of the influenza B viruses in the clinical specimens. RNA was extracted from clinical specimens by using the simplyRNA Tissue Kit (Promega) or RNeasy Mini Kit (Qiagen). Amplification and detection by real‐time PCR were performed with the Applied Biosystems 7900HT Fast Real‐Time PCR System (Applied Biosystems), the StepOnePlus Real‐Time PCR System (Applied Biosystems), or the LightCycler 96 System (Roche). RT‐PCR was carried out using the QuantiTect multiplex RT‐PCR kit (Qiagen) or QuantiTect Probe RT‐PCR Kit (Qiagen). The probes were designed to target the HA genes of B/Yamagata‐ or B/Victoria‐lineage viruses. The probes contained oligonucleotides with the 6‐carboxyfluorescein (FAM) or the hexacholoro‐6‐carboxyfluorescein (HEX) reporter dye at the 5′ end and the Black Hole Quencher‐1 (BHQ‐1) or 6‐carboxytetramethylrhodamine (TAMRA) quencher dye at the 3′ end. A list of the primers and probes used is provided in Table S1.

### 2.6 RT‐PCR and sequencing of viral genes

Viral RNA was extracted from infected cell culture supernatant by using the QIAmp Viral RNA Mini Kit (Qiagen) and reverse‐transcribed to cDNA by using Superscript III reverse transcriptase (Thermo Fisher Scientific) and the Uni9/FluB1 primer.^12^ PCR was performed with primers specific for the HA or NA genes of influenza B virus. The PCR products were purified and directly sequenced by using BigDye Terminator version 3.1 Cycle Sequencing Kits (Thermo Fisher Scientific), and were then analyzed on an ABI Prism 3130xl Genetic Analyzer (Thermo Fisher Scientific). A list of the primers and probes used is provided in Table S1. The nucleotide sequences obtained in this study were submitted to GISAID's EpiFlu™ Database and assigned accession numbers as documented in Table S2.

### 2.7 Phylogenic analysis

Nucleotide sequences of the HA genes of influenza B viruses were aligned using mega version 7.0.26.^13^ The phylogenic tree of the HA nucleotide sequences was built in maximum‐likelihood method with 1000 bootstrap replicates using mega version 7.0.26.^13^

### 2.8 Experimental infection of ferrets

Six‐ to seven‐month‐old female ferrets (Wuxi Sangosho Biotechnology Co., Ltd.) were used in this study. Ferrets were anesthetized intramuscularly with ketamine and xylazine (5‐30 mg and 0.2‐6 mg/kg of body weight, respectively) and inoculated intranasally with 10^6^ PFU (0.5 mL) of B/Yokohama/5/2004, B/Brisbane/60/2008, B/Wisconsin/01/2010, B/Massachusetts/02/2012, B/Phuket/3073/2013, B/Texas/02/2013, or B/Colorado/06/2017. These ferrets did not receive a booster immunization. Serum samples were collected and subjected to the hemagglutination inhibition (HI) assay. All experiments with ferrets were performed in accordance with the University of Tokyo's Regulations for Animal Care and Use and were approved by the Animal Experiment Committee of the Institute of Medical Science, the University of Tokyo.

### 2.9 HI assay

Ferret sera were treated with receptor‐destroying enzyme (RDE II; Denka Seiken Co., Ltd) at 37°C for 20 hours, followed by RDE inactivation at 56°C for 30‐60 minutes. The treated sera were serially diluted twofold with PBS in 96‐well U‐bottom microtiter plates and mixed with the amount of virus equivalent to four hemagglutination units, followed by incubation at room temperature (25°C) for 60 minutes. After addition of 50 µL of 0.5% chicken red blood cells, the mixtures were gently mixed and incubated at 4°C for a further 45 minutes. HI titers are expressed as the inverse of the highest antibody dilution that inhibited hemagglutination.

### 2.10 NA inhibition assay

Oseltamivir carboxylate, peramivir, and laninamivir were kindly provided by Daiichi Sankyo Inc, Tokyo, Japan. Zanamivir was obtained from GlaxoSmithKline, London, UK. In vitro NA activity of viruses was determined as described previously.^14^, ^15^ Briefly, 2′‐(4‐methylumbelliferyl)‐α‐D‐N‐acetylneuraminic acid (MUNANA; Sigma, St Louis, Mo) at a final concentration of 0.1 mmol/L was used as a fluorescent substrate. Dilutions of viruses containing NA activity equivalent to 800‐1200 fluorescence units were used in this study. Ten microliters of the diluted viruses and 10 µL of the neuraminidase inhibitor (0.01 nmol/L to 10 µmol/L) in calcium‐MES buffer (33 mmol/L 2‐[N‐morpholino]ethanesulfonic acid, 4 mmol/L CaCl_2_, pH 6.0) were mixed and incubated at 37°C for 30 minutes, followed by the addition of 30 µL of the substrate. The mixtures were further incubated at 37°C for 1 hour, and the reaction was stopped by adding 150 µL of 0.1 mol/L sodium hydroxide in 80% ethanol (pH 10.0). The fluorescence of the solution was measured at an excitation wavelength of 360 nm and an emission wavelength of 465 nm. The relationship between the concentration of inhibitor and the percentage of fluorescence inhibition was determined, and 50% inhibitory concentration (IC_50_) values were obtained by extrapolating those findings. The IC_50_ values were calculated according to the IC_50_ analysis protocol provided by the Health Protection Agency, London, UK.^16^

## 3 RESULTS

### 3.1 Detection of influenza B viruses in Japan during the 2017‐2018 and 2018‐2019 seasons

During the 2017‐2018 and 2018‐2019 seasons, a total of 554 respiratory specimens were collected from patients with an influenza‐like illness in Tokyo, Japan, and 108 were confirmed to be influenza B virus‐positive by real‐time RT‐PCR using lineage‐specific primers/probes. Of 97 influenza B virus‐positive samples collected during the 2017‐2018 season, 95 (97.9%) belonged to the B/Yamagata‐lineage and two (2.1%) to the B/Victoria‐lineage. These results are consistent with the National Institute of Infectious Diseases (NIID) report that influenza B viruses of the B/Yamagata‐ and B/Victoria‐lineages co‐circulated in Japan during the winter season with the B/Yamagata‐lineage predominating.^7^ Of 11 influenza B virus‐positive samples collected during the 2018‐2019 season, two were of the B/Yamagata‐linage and nine were of the B/Victoria‐lineage. All 108 influenza B viruses were isolated from the clinical samples by inoculation to MDCK, AX4, or hCK cells. Of the 108 influenza B isolates, 68 (62 from the 2017‐2018 season and 6 from the 2018‐2019 season) were selected for further characterization.

### 3.2 Antigenic and genetic characterization of influenza B viruses isolated in Japan during the 2017‐2018 and 2018‐2019 seasons

**Figure 1:**
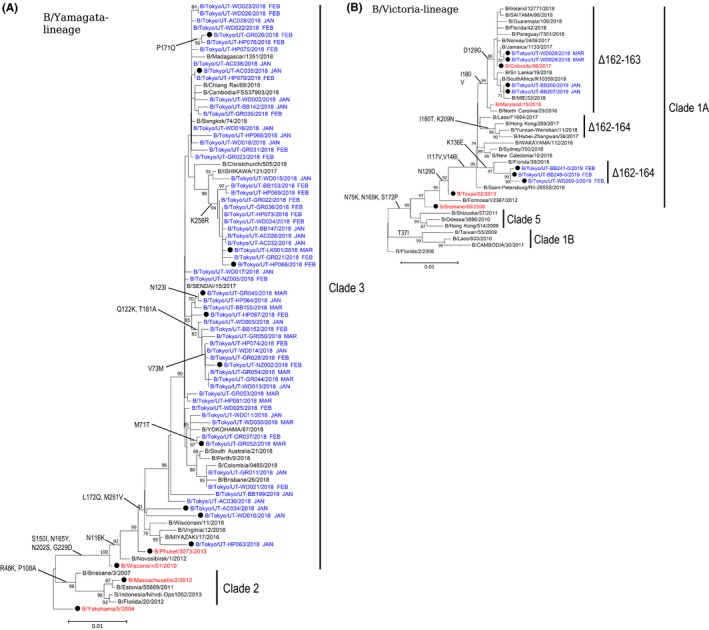
Phylogenic tree of the HA genes of (A) B/Yamagata‐ and (B) B/Victoria‐lineage viruses isolated in Japan during the 2017‐2018 and 2018‐2019 influenza seasons. The tree was built using the neighbor‐joining method with 1000 bootstrap replicates. Bootstrap values ≥ 70 are displayed on the branches. The month during which the clinical specimens were collected is shown after each strain name. The scale bar shows a 1% nucleotide change between close relatives. A and B, Influenza B isolates examined in this study are shown in blue. Vaccine and vaccine‐related strains are indicated in red. The black circle symbol indicated the viruses that were subjected to the HI assay. B, The Δ162‐163 and Δ162‐164 indicate viruses possessing the two‐amino‐acid deletion at positions 162 and 163 in HA and viruses possessing the three‐amino‐acid deletion at positions 162‐164 in HA, respectively

Recent B/Yamagata‐lineage viruses have been classified into two distinct genetic clades based on the phylogenetic characterization of the HA gene: clade 2 and clade 3, represented by the vaccine reference strains B/Massachusetts/2/2012 and B/Wisconsin/01/2010, respectively.^17^ In contrast, recent B/Victoria‐lineage viruses belong to clade 1A, which is represented by the vaccine reference strain B/Brisbane/60/2008. To determine the HA gene clade of the 68 influenza B viruses isolated in Japan during the 2017‐2018 and 2018‐2019 seasons, we performed phylogenetic analysis of their HA genes together with those of other viruses from the Global Initiative on Sharing Avian Influenza Data (GISAID) EpiFlu database. Since the 2015‐2016 season, only quadrivalent influenza vaccines containing two influenza A virus strains (A/H1N1pdm and A/H3N2) and two influenza B virus strains (B/Yamagata‐lineage and B/Victoria‐lineage viruses) have been used in Japan (Table S3). This analysis showed that all 61 B/Yamagata‐lineage virus isolates fell into clade 3 along with the vaccine strain B/Phuket/3073/2013, which was used in Japan for the 2017‐2018 and 2018‐2019 seasons (Figure 1A and Table S3). The seven B/Victoria‐lineage isolates possessed HA genes that belonged to clade 1A along with the vaccine strains B/Texas/02/2013 (B/Brisbane/60/2008‐like virus) and B/Maryland/15/2016 (B/Colorado/06/2017‐like virus), which were used in Japan for the 2017‐2018 and the 2018‐2019 seasons, respectively (Figure 1B and Table S3). B/Colorado/06/2017 contains a two‐amino‐acid deletion at positions 162 and 163 in its HA. Two isolates of the 2017‐2018 season and two isolates of the 2018‐2019 season had this two‐amino‐acid deletion. The remaining three isolates of the 2018‐2019 season harbored a three‐amino‐acid deletion at positions 162‐164 of HA. B/Victoria‐lineage viruses with the three‐amino‐acid deletion fell into two distinct subgroups (Figure 1B): one subgroup shared an amino acid substitution at position K136E, and the other subgroup had two common amino acid substitutions at I180T and K209N. All three viruses with the three‐amino‐acid deletion that were isolated during the 2018‐2019 season in Japan belonged to the former subgroup.

To elucidate whether influenza B viruses isolated in Japan during the 2017‐2018 and 2018‐2019 influenza seasons were antigenically related to the vaccine strains used for these two seasons in Japan, we performed an HI assay using post‐infection ferret antisera raised against cell culture‐propagated vaccine or vaccine‐related strains. For the B/Yamagata‐lineage viruses, all 11 isolates tested, which had amino acid substitutions in the globular head domain of their HA compared with the B/Phuket/3073/2013 virus, were readily recognized by the ferret antisera raised against the cell culture‐propagated vaccine viruses B/Wisconsin/01/2010 and B/Phuket/3073/2013 at titers within twofold of the homologous titer of these antisera and were less well recognized by the ferret antisera raised against the cell culture‐propagated vaccine virus B/Massachusetts/2/2012 (titers within twofold or fourfold of the homologous titer) (Table 1). However, like the clade 3 reference viruses B/Wisconsin/01/2010 and B/Phuket/3073/2013, these 11 isolates were poorly recognized by the ferret antisera raised against cell culture‐propagated B/Yokohama/5/2004, which is genetically closely related to the vaccine virus B/Florida/4/2006, with titers 16‐ or 32‐fold lower than the homologous titer. These results indicate that all tested B/Yamagata‐lineage isolates were antigenically and genetically closely related to the vaccine strains B/Wisconsin/01/2010 and B/Phuket/3073/2013.

**Table 1:** Antigenic analysis of B/Yamagata‐lineage viruses isolated in Japan during the 2017‐2018 season

Virus	Passage history	Genetic clade	Hemagglutination inhibition titer			
			Ferret antiserum			
			B/Yokohama/5/2004	B/Wisconsin/01/2010	B/Massachusetts/2/2012	B/Phuket/3073/2013
Reference viruses						
B/Yokohama/5/2004	AX4‐X^a^ + MDCK2	1	**1280**	640	640	320
B/Wisconsin/01/2010	MDCK5	3	80	**640**	320	320
B/Massachusetts/2/2012	MDCK6	2	640	320	**640**	160
B/Phuket/3073/2013	MDCK5	3	80	320	320	**320**
Test viruses						
B/Tokyo/UT‐AC034/2018	MDCK2	3	80	320	320	320
B/Tokyo/UT‐AC035/2018	MDCK2	3	80	320	160	160
B/Tokyo/UT‐GR026/2018	MDCK2	3	80	320	160	160
B/Tokyo/UT‐GR045/2018	MDCK2	3	40	320	160	160
B/Tokyo/UT‐GR052/2018	MDCK2	3	40	320	160	160
B/Tokyo/UT‐HP063/2018	MDCK2	3	80	320	320	320
B/Tokyo/UT‐HP067/2018	MDCK2	3	80	320	320	320
B/Tokyo/UT‐HP068/2018	MDCK2	3	80	320	320	160
B/Tokyo/UT‐NZ002/2018	MDCK2	3	80	640	320	320
B/Tokyo/UT‐LK001/2018	AX4‐1 + MDCK1	3	80	320	320	160
B/Tokyo/UT‐WD010/2018	MDCK2	3	80	640	320	320

Note

Homologous titers are underlined and bolded.

a X, unknown passage number.

For B/Victoria‐lineage viruses, all 7 isolates were subjected to an HI assay. All 7 of the tested isolates were poorly recognized by the ferret antiserum raised against the cell culture‐propagated vaccine strain B/Texas/02/2013, with titers eightfold or 16‐fold lower than the homologous titer of this antiserum (Tables 2 and 3). An antiserum raised against the cell culture‐propagated vaccine strain B/Brisbane/60/2008 recognized the 7 isolates but at fourfold, eightfold, or 16‐fold reduced levels compared with the titers of the antiserum for the homologous virus. In contrast, antisera raised against cell culture‐propagated B/Colorado/06/2017 efficiently inhibited the hemagglutination activity of the four test viruses lacking two amino acids in their HAs, with titers similar to that of the homologous virus*,* consistent with the WHO report.^18^ However, the three test viruses carrying the three‐amino‐acid deletion were recognized less well by the antiserum against B/Colorado/06/2017 with titers fourfold or eightfold lower than the homologous titer of this antiserum. These results indicate that the four test viruses with the two‐amino‐acid deletion are antigenically and genetically closely related to the vaccine virus B/Colorado/06/2017, but the remaining three test viruses with the three‐amino‐acid deletion may be antigenically different from the vaccine strain.

**Table 2:** Antigenic analysis of B/Victoria‐lineage viruses isolated in Japan during the 2017‐2018 season

Virus	Passage history	Genetic clade	Hemagglutination inhibition titer		
			Ferret antiserum		
			B/Brisbane/60/2008	B/Texas/02/2013	B/Colorado/06/2017
Reference viruses					
B/Brisbane/60/2008	MDCKX^a^	1A	**2560**	1280	320
B/Texas/02/2013	MDCK6	1A	1280	**1280**	320
B/Colorado/06/2017	MDCK4	1A (Δ2 aa)	640	160	**1280**
Test viruses					
B/Tokyo/UT‐WD028/2018	MDCK2	1A (Δ2 aa)	160	80	1280
B/Tokyo/UT‐WD029/2018	MDCK2	1A (Δ2 aa)	320	160	1280

Note

Homologous titers are underlined and bolded.

a X, unknown passage number.

**Table 3:** Antigenic analysis of B/Victoria‐lineage viruses isolated in Japan during the 2018‐2019 season

Virus	Passage history	Genetic clade	Hemagglutination inhibition titer		
			Ferret antiserum		
			B/Brisbane/60/2008	B/Texas/02/2013	B/Colorado/06/2017
Reference viruses					
B/Brisbane/60/2008	MDCKX^a^	1A	**2560**	640	320
B/Texas/02/2013	MDCK6	1A	1280	**1280**	160
B/Colorado/06/2017	MDCK4	1A (Δ2 aa)	640	160	**1280**
Test viruses					
B/Tokyo/UT‐BB200/2019	hCK2	1A (Δ2 aa)	640	80	1280
B/Tokyo/UT‐BB207/2019	hCK2	1A (Δ2 aa)	640	160	1280
B/Tokyo/UT‐BB241‐0/2019	hCK2	1A (Δ3 aa)	640	80	320
B/Tokyo/UT‐BB248‐0/2019	hCK2	1A (Δ3 aa)	320	80	160
B/Tokyo/UT‐WD050‐0/2019	hCK2	1A (Δ3 aa)	320	80	160

Note

Homologous titers are underlined and bolded.

a X, unknown passage number.

### 3.3 Antiviral susceptibility

To monitor the susceptibility of influenza B viruses to NA inhibitors in Japan during the 2017‐2018 and 2018‐2019 seasons, the nucleotide sequences of the NA segments of the 68 isolates were determined by means of Sanger sequencing. Sequence analysis revealed no mutations known to confer resistance to NA inhibitors in the influenza B isolates, except for one B/Yamagata‐lineage isolate (B/Tokyo/UT‐AC032/2018) that possessed a G407S mutation in its NA.^15^ A fluorescence NA inhibition assay with the MUNANA substrate was used to characterize the susceptibility of B/Tokyo/UT‐AC032/2018 virus to oseltamivir carboxylate, peramivir, zanamivir, and laninamivir (Table 4). The isolate showed a marked reduction in susceptibility to peramivir, zanamivir, and laninamivir (64‐, 167‐, and 204‐fold increases in IC_50_ values, respectively, compared with a drug‐susceptible control virus, a recombinant virus possessing the HA and NA genes from B/Phuket/3073/2013). This isolate also exhibited moderately reduced susceptibility to oseltamivir carboxylate (eightfold).

**Table 4:** Virus sensitivity to NA inhibitors *in vitro* a

Virus	NA change	IC_50_ values (nM) of NA inhibitors (fold differences^b^)			
		Oseltamivir carboxylate^c^	Zanamivir	Peramivir	Laninamivir^d^
rPhuket3073/Yamagata^e^	wild‐type	64.9 ± 11.7 (1.0)	5.9 ± 1.1 (1.0)	0.5 ± 0.1 (1.0)	2.5 ± 0.1 (1.0)
rPhuket3073/Yamagata‐E117A^f^	E117A	38 075.9 ± 7365.0 (586.6)	164 197.3 ± 29 976.2 (27 830.0)	14 959.5 ± 296.9 (29 919.0)	54 022.7 ± 4659.7 (21 609.1)
B/Tokyo/UT‐AC032/2018	G407S	537.2 ± 215.7 (8.3)	986.6 ± 507.9 (167.2)	32.1 ± 12.9 (64.2)	510.1 ± 244.4 (204.0)

a IC_50_ values were determined by using an NA‐Fluor Influenza Neuraminidase Assay Kit. Average IC_50_ values and standard deviations were calculated from three independent assays performed in duplicate.

b Compared with IC_50_ values obtained with the sensitive control rPhuket3073/Yamagata.

c Oseltamivir carboxylate is the active form of oseltamivir.

d Laninamivir is the active form of laninamivir octanoate.

e A recombinant virus possessing the HA and NA genes from B/Phuket/3073/2013 and the remaining genes from B/Yamagata/1/73 virus.

f A recombinant Phuket3073/Yamagata virus with an E117A mutation in its NA.

## 4 DISCUSSION

Most of the B/Victoria‐lineage viruses that were detected worldwide during the eight influenza seasons that followed the 2009‐2010 season were antigenically closely related to the vaccine strain B/Brisbane/60/2008.^19^ In this study, during the 2017‐2018 and/or 2018‐2019 seasons in Japan, we detected B/Victoria‐lineage viruses with a deletion of two or three amino acids at positions 162 and 163 or 162‐164 in HA, respectively, which are antigenically distinct from the vaccine strain. Antigenic variants possessing the two‐amino‐acid deletion were isolated in the United States during the 2016‐2017 season,^20^ whereas variants with the three‐amino‐acid deletion were isolated in China and Hong Kong during the 2017‐2018 season.^21^ Subsequently, these antigenic variants have been reported in many countries.^18^, ^22^ The WHO recommended the B/Colorado/06/2017‐like virus with the two‐amino‐acid deletion for the 2019‐2020 northern hemisphere influenza vaccine.^18^ Our antigenic analyses with post‐infection ferret sera showed that all tested B/Victoria‐lineage viruses with the three‐amino‐acid deletion were inhibited less well by the antiserum raised against B/Colorado/06/2017 than viruses that possess the two‐amino‐acid deletion (Table 3), consistent with the WHO report.^18^ These findings suggest that contemporary B/Victoria‐lineage viruses with the three‐amino‐acid deletion may be antigenically distinguishable from viruses with the two‐amino‐acid deletion. Importantly, an increasing proportion of a variant with the three‐amino‐acid deletion was reported in many countries during the 2018‐2019 season.^18^ Therefore, continued monitoring for the prevalence of B/Victoria‐lineage viruses with the three‐amino‐acid deletion is important.

Influenza B viruses carrying the G407S mutation in their NA, which confers reduced susceptibility to NA inhibitors, were sporadically detected in Japan during the 2004‐2005 season^15^ and in Australia and the United States during the 2015‐2016 season.^23^ In this study, we also detected an influenza B virus with the NA‐G407S mutation during the 2017‐2018 season in Japan (Table 4). This virus (B/Tokyo/UT‐AC032/2018) was isolated from a specimen collected from an adult patient (a 41‐year‐old female) prior to drug treatment. Although it is unclear whether the patient had contact with an influenza patient who was treated with NA inhibitors, it is possible that this variant may be transmissible from person to person. Further investigations are required to determine whether this NA‐G407S mutation affects the transmissibility of influenza B viruses.

This virological surveillance study may have been limited by the fact that samples were collected in a limited area in Japan and by the relatively small number of influenza B isolates. Nevertheless, such monitoring of the most recent circulating strains contributes to the early detection of new antigenic variants and drug‐resistant viruses.

## CONFLICT OF INTEREST

SK‐M., YS‐T., KI‐H., M.Ito., AT, HH, NI, TN, NW, MK, EA, DJ, HY, and M. Imai have no competing interests. MY has received speaker's honoraria from Daiichi Sankyo Co., Ltd. YK has received speaker's honoraria from Toyama Chemical and Astellas, Inc; grant support from Daiichi Sankyo Pharmaceutical, Toyama Chemical, Shionogi & Co., Ltd., and Kyoritsu Seiyaku; and is a founder of FluGen.
