# Supplementary material for: Antigenic variants of influenza B viruses isolated in Japan during the 2017‐2018 and 2018‐2019 influenza seasons
Source: Influenza Other Respir Viruses. 2020 Jan 19;14(3):311–9. doi: 10.1111/irv.12713 (PMC7182600; doi:10.1111/irv.12713)
Supplement: Supplementary file 1 [file IRV-14-311-s001.docx]

**Supporting Information**

**Antigenic variants of influenza B viruses isolated in Japan during the 2017–2018 and 2018–2019 influenza seasons**

Sari Kato-Miyashita, Yuko Sakai-Tagawa, Makoto Yamashita, Kiyoko Iwatsuki-Horimoto, Mutsumi Ito, Akifumi Tokita, Haruhisa Hagiwara, Naomi Izumida, Tamon Nishino, Noriyuki Wada, Michiko Koga, Eisuke Adachi, Daisuke Jubishi, Hiroshi Yotsuyanagi**,** Yoshihiro Kawaoka**,** Masaki Imai.

**Table S1. List of primers and probes used in this study**

| Primer or probe | Target gene | Sequence (5' - 3')^†^ | Orientation |
| --- | --- | --- | --- |
| BHA1-N | Influenza B virus HA | AATATCCACAAAATGAAGGC | Forward |
| BHA2-1867-1887R | Influenza B virus HA | AGTAGTAACAAGAGCATTTTT | Reverse |
| BNA-F5v2 | Influenza B virus NA | TCAAAACTGAAGCAAATAGGCCA | Forward |
| BNA-R1498-1472 | Influenza B virus NA | AATAGGAACAAAGGGTTTAGAACAGA | Reverse |
| qPCR-B/Victoria-HA-F | B/Victoria virus HA | CCTGTTACATCTGGGTGCTTTCCTATAATG | Forward |
| qPCR-B/Victoria-HA-R | B/Victoria virus HA | GTTGATARCCTGATATGTTCGTATCCTCKG | Reverse |
| qPCR-B/Yamagata-HA-F | B/Yamagata virus HA | CCTGTTACATCCGGGTGCTTYCCTATAATG | Forward |
| qPCR-B/Yamagata-HA-R | B/Yamagata virus HA | GTTGATAACCTKATMTTTTCATATCCTCTG | Reverse |
| FAM-B/Victoria-HA-Probe | B/Victoria virus HA | (FAM)TTAGACAGCTGCCTAACC(BHQ-1) |  |
| FAM-Type B HA Victoria | B/Victoria virus HA | (FAM)TTAGACAGCTGCCTAACC(MGB/TAMRA) |  |
| HEX-B/Yamagata-HA-Probe | B/Yamagata virus HA | (HEX)TCAGGCAACTASCCAATC(BHQ-1) |  |
| FAM-Type B HA Yamagata | B/Yamagata virus HA | (FAM)TCAGGCAACTASCCAATC(MGB/TAMRA) |  |

^†^FAM, 6-carboxyfluorescein; HEX, hexacholoro-6-carboxyfluorescein; BHQ-1, black hole quencher; MGB, minor groove binder; TAMRA, 6-carboxytetramethylrhodamine.

**Table S2. GISAID accession numbers for influenza B viruses used in this study**

| Influenza B isolates examined in this study | GISAID accession numbers | | Reference strains | GISAID accession numbers |
| --- | --- | --- | --- | --- |
|  | HA gene | NA gene |  | HA gene |
| B/Tokyo/UT-AC026/2018 | EPI1590525 | EPI1590526 | B/Yokohama/5/2004 | EPI1590698 |
| B/Tokyo/UT-AC028/2018 | EPI1590514 | EPI1590515 | B/Wisconsin/01/2011 | EPI363743 |
| B/Tokyo/UT-AC030/2018 | EPI1590557 | EPI1590560 | B/Massachusetts/2/2012 | EPI368425 |
| B/Tokyo/UT-AC032/2018 | EPI1590558 | EPI1590561 | B/Phuket/3073/2013 | EPI540675 |
| B/Tokyo/UT-AC034/2018 | EPI1590559 | EPI1590562 | B/Madagascar/1351/2018 | EPI1281995 |
| B/Tokyo/UT-AC035/2018 | EPI1590563 | EPI1590564 | B/Chiang Rai/69/2018 | EPI1283071 |
| B/Tokyo/UT-AC038/2018 | EPI1590565 | EPI1590566 | B/Cambodia/FSS37903/2018 | EPI1283267 |
| B/Tokyo/UT-BB142/2018 | EPI1590567 | EPI1590568 | B/Bangkok/74/2018 | EPI1283073 |
| B/Tokyo/UT-BB147/2018 | EPI1590569 | EPI1590570 | B/Christchurch/505/2018 | EPI1283265 |
| B/Tokyo/UT-BB152/2018 | EPI1590571 | EPI1590572 | B/ISHIKAWA/121/2017 | EPI1260419 |
| B/Tokyo/UT-BB153/2018 | EPI1590573 | EPI1590574 | B/SENDAI/15/2017 | EPI1260433 |
| B/Tokyo/UT-BB155/2018 | EPI1590575 | EPI1590576 | B/YOKOHAMA/87/2018 | EPI1260427 |
| B/Tokyo/UT-GR011/2018 | EPI1590577 | EPI1590578 | B/South Australia/21/2018 | EPI1283267 |
| B/Tokyo/UT-GR021/2018 | EPI1590579 | EPI1590580 | B/Perth/9/2018 | EPI1283271 |
| B/Tokyo/UT-GR022/2018 | EPI1590581 | EPI1590582 | B/Colombia/0485/2018 | EPI1260433 |
| B/Tokyo/UT-GR023/2018 | EPI1590583 | EPI1590584 | B/Brisbane/26/2018 | EPI1279553 |
| B/Tokyo/UT-GR026/2018 | EPI1590585 | EPI1590586 | B/Wisconsin/11/2016 | EPI745461 |
| B/Tokyo/UT-GR028/2018 | EPI1590587 | EPI1590588 | B/Virginia/12/2016 | EPI745405 |
| B/Tokyo/UT-GR031/2018 | EPI1590589 | EPI1590590 | B/MIYAZAKI/17/2016 | EPI740476 |
| B/Tokyo/UT-GR035/2018 | EPI1590591 | EPI1590592 | B/Novosibirsk/1/2012 | EPI904265 |
| B/Tokyo/UT-GR036/2018 | EPI1590593 | EPI1590594 | B/Brisbane/3/2007 | EPI483156 |
| B/Tokyo/UT-GR037/2018 | EPI1590595 | EPI1590596 | B/Estonia/55669/2011 | EPI904353 |
| B/Tokyo/UT-GR044/2018 | EPI1590597 | EPI1590598 | B/Indonesia/Nihrdi-Dps1052/2013 | EPI498321 |
| B/Tokyo/UT-GR045/2018 | EPI1590599 | EPI1590600 | B/Florida/20/2012 | EPI391315 |
| B/Tokyo/UT-GR050/2018 | EPI1590601 | EPI1590602 | B/Brisbane/60/2008 | EPI283743 |
| B/Tokyo/UT-GR052/2018 | EPI1590603 | EPI1590604 | B/Texas/02/2013 | EPI418192 |
| B/Tokyo/UT-GR053/2018 | EPI1590605 | EPI1590606 | B/Colorado/06/2017 | EPI1055528 |
| B/Tokyo/UT-GR054/2018 | EPI1590607 | EPI1590608 | B/Florida/2/2012 | EPI1590697 |
| B/Tokyo/UT-HP063/2018 | EPI1590609 | EPI1590610 | B/Florida/42/2018 | EPI1273609 |
| B/Tokyo/UT-HP064/2018 | EPI1590611 | EPI1590612 | B/Ireland/12771/2018 | EPI1277034 |
| B/Tokyo/UT-HP066/2018 | EPI1590613 | EPI1590614 | B/Paraguay/7301/2018 | EPI1278407 |
| B/Tokyo/UT-HP067/2018 | EPI1590615 | EPI1590616 | B/Norway/2409/2017 | EPI1010244 |
| B/Tokyo/UT-HP068/2018 | EPI1590617 | EPI1590618 | B/Guatemala/109/2018 | EPI1281829 |
| B/Tokyo/UT-HP069/2018 | EPI1590619 | EPI1590620 | B/Jamaica/1133/2017 | EPI1034029 |
| B/Tokyo/UT-HP070/2018 | EPI1590621 | EPI1590622 | B/SouthAfrica/R10359/2018 | EPI1277046 |
| B/Tokyo/UT-HP073/2018 | EPI1590623 | EPI1590624 | B/Sri Lanka/19/2018 | EPI1283075 |
| B/Tokyo/UT-HP074/2018 | EPI1590625 | EPI1590626 | B/North Carolina/29/2016 | EPI948705 |
| B/Tokyo/UT-HP075/2018 | EPI1590627 | EPI1590628 | B/Laos/F1664/2017 | EPI1113225 |
| B/Tokyo/UT-HP076/2018 | EPI1590629 | EPI1590630 | B/Hong Kong/269/2017 | EPI1052656 |
| B/Tokyo/UT-HP081/2018 | EPI1590631 | EPI1590632 | B/Yunnan-Wenshan/11/2018 | EPI1168596 |
| B/Tokyo/UT-NZ002/2018 | EPI1590633 | EPI1590634 | B/Hubei-Zhangwan/36/2017 | EPI1168599 |
| B/Tokyo/UT-NZ005/2018 | EPI1590635 | EPI1590636 | B/Sydney/700/2018 | EPI1283077 |
| B/Tokyo/UT-LK001/2018 | EPI1590637 | EPI1590638 | B/New Caledonia/10/2018 | EPI1283083 |
| B/Tokyo/UT-WD002/2018 | EPI1590639 | EPI1590640 | B/Saint-Petersburg/RII-2855S/2018 | EPI1280871 |
| B/Tokyo/UT-WD003/2018 | EPI1590641 | EPI1590642 | B/Formosa/V2367/2012 | EPI904348 |
| B/Tokyo/UT-WD010/2018 | EPI1590643 | EPI1590644 | B/Shizuoka/57/2011 | EPI340837 |
| B/Tokyo/UT-WD011/2018 | EPI1590645 | EPI1590646 | B/Odessa/3886/2010 | EPI902450 |
| B/Tokyo/UT-WD013/2018 | EPI1590647 | EPI1590648 | B/Hong Kong/514/2009 | EPI243627 |
| B/Tokyo/UT-WD014/2018 | EPI1590649 | EPI1590650 | B/Taiwan/55/2009 | EPI241199 |
| B/Tokyo/UT-WD015/2018 | EPI1590651 | EPI1590652 | B/Laos/833/2010 | EPI1113225 |
| B/Tokyo/UT-WD016/2018 | EPI1590653 | EPI1590654 | B/CAMBODIA/30/2011 | EPI331319 |
| B/Tokyo/UT-WD017/2018 | EPI1590655 | EPI1590656 | B/Maryland/06/2017 | EPI978309 |
| B/Tokyo/UT-WD018/2018 | EPI1590657 | EPI1590658 |  |  |
| B/Tokyo/UT-WD021/2018 | EPI1590659 | EPI1590660 |  |  |
| B/Tokyo/UT-WD022/2018 | EPI1590661 | EPI1590662 |  |  |
| B/Tokyo/UT-WD023/2018 | EPI1590663 | EPI1590664 |  |  |
| B/Tokyo/UT-WD024/2018 | EPI1590665 | EPI1590666 |  |  |
| B/Tokyo/UT-WD025/2018 | EPI1590667 | EPI1590668 |  |  |
| B/Tokyo/UT-WD026/2018 | EPI1590669 | EPI1590670 |  |  |
| B/Tokyo/UT-WD028/2018 | EPI1590686 | EPI1590689 |  |  |
| B/Tokyo/UT-WD029/2018 | EPI1590687 | EPI1590688 |  |  |
| B/Tokyo/UT-WD030/2018 | EPI1590671 | EPI1590672 |  |  |
| B/Tokyo/UT-BB199/2019 | EPI1590673 | EPI1590674 |  |  |
| B/Tokyo/UT-BB200/2019 | EPI1590690 | EPI1590675 |  |  |
| B/Tokyo/UT-BB207/2019 | EPI1590691 | EPI1590676 |  |  |
| B/Tokyo/UT-BB241-0/2019 | EPI1590692 | EPI1590677 |  |  |
| B/Tokyo/UT-BB248-0/2019 | EPI1590693 | EPI1590694 |  |  |
| B/Tokyo/UT-WD050-0/2019 | EPI1590695 | EPI1590696 |  |  |

**Table S3. Influenza B vaccine strains recommended by the WHO or approved for use in Japan**

| Season | Vaccine strains recommended by the WHO^†^ | Vaccine strains approved for use in Japan |
| --- | --- | --- |
| 2011–2012 | a B/Brisbane/60/2008-like virus (B/Victoria-lineage) | B/Brisbane/60/2008 (B/Victoria-lineage) |
| 2012–2013 | a B/Wisconsin/01/2010-like virus (B/Yamagata-lineage) | B/Wisconsin/01/2010 (B/Yamagata-lineage) |
| 2013–2014 | a B/Massachusetts/2/2012-like (B/Yamagata-lineage) and  a B/Brisbane/60/2008-like (B/Victoria-lineage) virus | B/Massachusetts/2/2012 (B/Yamagata-lineage) |
| 2014–2015 | a B/Massachusetts/2/2012-like (B/Yamagata-lineage) and  a B/Brisbane/60/2008-like (B/Victoria-lineage) virus | B/Massachusetts/2/2012 (B/Yamagata-lineage) |
| 2015–2016 | a B/Phuket/3073/2013-like (B/Yamagata-lineage) and  a B/Brisbane/60/2008-like (B/Victoria-lineage) virus | B/Phuket/3073/2013 (B/Yamagata-lineage) and B/Texas/02/2013 (B/Victoria-lineage) |
| 2016–2017 | a B/Phuket/3073/2013-like (B/Yamagata-lineage) and  a B/Brisbane/60/2008-like (B/Victoria-lineage) virus | B/Phuket/3073/2013 (B/Yamagata-lineage) and B/Texas/02/2013 (B/Victoria-lineage) |
| 2017–2018 | a B/Phuket/3073/2013-like (B/Yamagata-lineage) and  a B/Brisbane/60/2008-like (B/Victoria-lineage) virus | B/Phuket/3073/2013 (B/Yamagata-lineage) and B/Texas/02/2013 (B/Victoria-lineage) |
| 2018–2019 | a B/Phuket/3073/2013-like (B/Yamagata-lineage) and  a B/Colorado/06/2017-like (B/Victoria-lineage) virus | B/Phuket/3073/2013 (B/Yamagata-lineage) and B/Maryland/15/2016 (B/Victoria-lineage) |
| 2019–2020 | a B/Phuket/3073/2013-like (B/Yamagata-lineage) and  a B/Colorado/06/2017-like (B/Victoria-lineage) virus | B/Phuket/3073/2013 (B/Yamagata-lineage) and B/Maryland/15/2016 (B/Victoria-lineage) |

^†^for use in the Northern hemisphere influenza seasons
